# Supplementary material for: Characterization of the TREM-1 signaling landscape in human neutrophils
Source: PLoS One. 2026 Mar 5;21(3):e0334007. doi: 10.1371/journal.pone.0334007 (PMC12962488; doi:10.1371/journal.pone.0334007)
Supplement: Attachment — Submitted filename: Response to Reviewers.pdf [file pone.0334007.s002.pdf]

Response to Reviewers – PONE-D-25-47347

Point by point reply

Dear Academic Editor and Reviewers,

We would like to thank the Academic Editor and the reviewers for their careful evaluation of our manuscript entitled

“Characterization of the TREM-1 Signaling Landscape in Human Neutrophils” (PONE-D-25-47347).

We appreciate the constructive comments and believe that the revisions have substantially improved the clarity, transparency, and scientific rigor of the manuscript.

Below, we provide a detailed, point-by-point response to all editorial and reviewer comments. All changes have been incorporated into the revised manuscript and are highlighted in the version submitted with track changes.

Editorial Requirements

1. PLOS ONE style requirements

Response:
The manuscript has been reformatted according to the official PLOS ONE templates for the main text, title page, and author affiliations. File naming conventions have also been adjusted to comply with journal requirements.

Manuscript change:
Formatting updated throughout the manuscript.

---

2. Participant consent

Response:
We have expanded the ethics statement in the Methods section to explicitly state that informed consent was obtained from all participants and that consent was obtained in written form. No minors were included in this study.

Manuscript change:
Methods section, Ethics statement.

---

3. Role of the funders

Response:
We confirm that the funders had no role in study design, data collection and analysis, decision to publish, or preparation of the manuscript. This statement has been added to the cover letter as requested.

Cover letter addition:
"The funders had no role in study design, data collection and analysis, decision to publish, or preparation of the manuscript."

---

4. Data availability

Response:
We have updated the Data Availability Statement to clarify that all data underlying the findings are now deposited in publicly accessible repositories. Accession numbers and links are provided in the revised manuscript.

Manuscript change:
Data Availability section.

---

5. Use of "data not shown"

Response:
All instances of the phrase "data not shown" have been removed. Where relevant, corresponding data have either been included in the manuscript, added as Supporting Information, or deposited in a public repository with appropriate references.

Manuscript change:
Results and Discussion sections.

---

6. Placement of ethics statement

Response:
The ethics statement now appears exclusively in the Methods section and has been removed from all other sections.

Manuscript change:
Methods section.

---

7. Reviewer-recommended citations

Response:
All suggested references were carefully evaluated and cited where relevant to the revised manuscript.

Reviewer #1

Comment 1: Donor characteristics and sample size

Response:
We agree that donor characteristics are important for reproducibility. We have expanded the Methods section to include age range and sex distribution of donors. We acknowledge the limited number of donors as a limitation inherent to studies using primary human neutrophils and have explicitly addressed this point in the Discussion.

Manuscript change:
Methods (Donors) and Discussion (Limitations).

Comment 2: Typographical inconsistencies in cell numbers

Response:
We thank the reviewer for pointing this out. All reported cell numbers have been carefully reviewed and corrected for consistency and accuracy.

Manuscript change:
Methods section.

Comment 3: Annotation of Figure 1

Response:
Key kinases have now been explicitly labeled in Figure 1 (a–d) to improve readability and highlight relevant signaling components.

Manuscript change:
Figure 1 and corresponding legend.

Comment 4: Overlap between TREM-1 and TLR4 signaling

Response:
We agree that LPS can indirectly amplify TREM-1 signaling. This conceptual

limitation is now explicitly discussed. Our intention was not to claim complete separation of TLR4- and TREM-1-mediated pathways, but rather to identify shared and distinct signaling tendencies. We have clarified this rationale and interpretation in the Discussion.

Manuscript change:
Discussion section.

Comment 5: Font size in Figure 3

Response:
Font sizes in Figure 3 (a–d) have been increased to improve readability.

Manuscript change:
Figure 3.

Comment 6: Dissecting TLR4 versus TREM-1 signaling

Response:
We acknowledge that genetic knockdown approaches would be required for definitive pathway separation. However, primary human neutrophils are not amenable to stable genetic manipulation. We have clarified this methodological limitation and framed our conclusions accordingly.

Manuscript change:
Discussion (Limitations).

Comment 7: Lack of functional assessment

Response:
We agree that functional validation would further strengthen the study. The current work was designed as a comprehensive signaling and transcriptomic characterization. We now clearly state this scope and limitation and outline future directions for functional validation.

Manuscript change:
Discussion section.

Reviewer #2

Comment 1: Inclusion of patient-derived PMNs or TANs

Response:
We agree that patient-derived cells would be highly informative. However, this study was intentionally designed to establish a baseline signaling landscape in healthy human neutrophils. This rationale and limitation are now explicitly discussed.

Manuscript change:
Discussion section.

Comment 2: Validation of kinome targets

Response:
We acknowledge this point and now emphasize that the kinome analysis provides a hypothesis-generating framework. Functional validation of selected targets is an important next step and is highlighted as future work.

Manuscript change:
Discussion section.

Concluding remarks

We believe that the revised manuscript addresses all concerns raised by the editor and reviewers and that it now meets the publication criteria of *PLOS ONE*. We sincerely thank the editor and reviewers for their insightful comments and the opportunity to revise our work.

Kind regards,

Markus P. Radsak
on behalf of all authors
